# Does Mineralocorticoid Receptor Antagonism Prevent Calcineurin Inhibitor-Induced Nephrotoxicity?

**DOI:** 10.3389/fmed.2017.00210

**Published:** 2017-11-24

**Authors:** Line Aas Mortensen, Claus Bistrup, Helle Charlotte Thiesson

**Affiliations:** ^1^Department of Nephrology, Odense University Hospital, Odense, Denmark; ^2^Department of Clinical Research, University of Southern Denmark, Odense, Denmark

**Keywords:** aldosterone, mineralocorticoid, cyclosporine A, tacrolimus, IF/TA, fibrosis

## Abstract

Calcineurin inhibitors have markedly reduced acute rejection rates in renal transplantation, thus significantly improved short-term outcome. The beneficial effects are, however, tampered by acute and chronic nephrotoxicity leading to interstitial fibrosis and tubular atrophy, which impairs long-term allograft survival. The mineralocorticoid hormone aldosterone induces fibrosis in numerous organs, including the kidney. Evidence from animal models suggests a beneficial effect of aldosterone antagonism in reducing calcineurin inhibitor-induced nephrotoxicity. This review summarizes current evidence of mineralocorticoid receptor antagonism in animal models of calcineurin inhibitor-induced nephrotoxicity and the results from studies of mineralocorticoid antagonism in renal transplant patients.

## Introduction

The introduction of calcineurin inhibitors (CNIs) in renal transplantation has markedly reduced the occurrence of acute rejection and graft failure (1, 2). The immunosuppressive effect of CNI is, however, counterbalanced by adverse long term effects on kidney function leading to histological changes (i.e., arteriolar hyalinosis, striped interstitial fibrosis, tubular atrophy, and glomerulosclerosis) referred to as interstitial fibrosis and tubular atrophy (IF/TA) (3).

The mineralocorticoid hormone aldosterone stimulates fibrogenesis in multiple organs. Evidence from animal and human studies indicates that antagonizing aldosterone signaling *via* the mineralocorticoid receptor (MR) by MR antagonists reduces CNI nephrotoxicity.

This review summarizes current evidence from animal studies and outlines the potential benefits of MR antagonism in kidney transplant patients.

## Calcineurin Inhibitor-Induced Nephrotoxicity

Although structurally different, both cyclosporine (CsA) and tacrolimus exert their effect by inhibiting the activity of calcineurin, a calcium- and calmodulin-dependent phosphatase involved in the activation of T-lymphocytes. Complexes of CsA/cyclophylin or tacrolimus/FKBP12 bind competitively to calcineurin, thereby preventing the dephosphorylation and subsequent activation of nuclear factor of activated T-cells (NFAT). Activated NFAT promotes transcription of interleukin-2, which is pivotal for the activation of T-lymphocytes (4).

**Figure 1:**
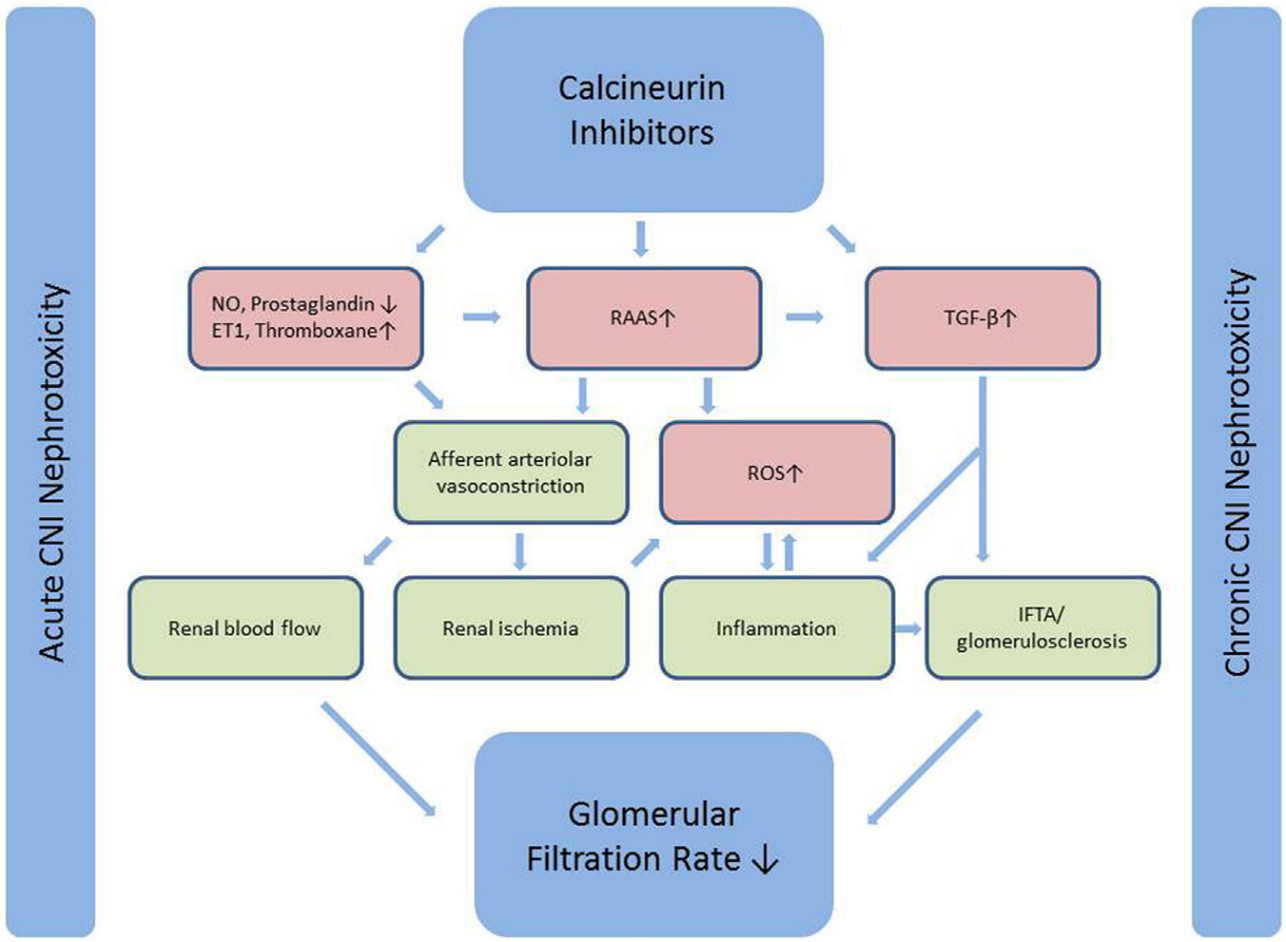
Calcineurin inhibitors induce afferent arteriolar vasoconstriction through an effect on both mediators of endothelial dysfunction and a direct stimulatory effect on the RAAS-system. Vasoconstriction leads to reduced renal blood flow (acute CNI nephrotoxicity) and renal ischemia, which ultimately leads to inflammation and fibrosis (chronic CNI nephrotoxicity). The latter is further induced by a direct stimulatory effect on the major pro-fibrotic cytokine TGF-β. Simplified from Naesens et al. (3). CNI, calcineurin inhibitor; NO, nitric oxide; ET1, endothelin 1; RAAS, renin-angiotensin-aldosterone system; TGF-β, transforming growth factor β; ROS, reactive oxygen species; IF/TA, interstitial fibrosis and tubular atrophy.

Nephrotoxicity has long been recognized as an adverse effect of CNI leading to chronic allograft failure and ultimately increased morbidity and mortality, mainly due to cardiovascular disease (5). Acute CNI nephrotoxicity is induced by vasoconstriction due to an imbalance between vasodilating and vasoconstricting factors and is reversible, whereas chronic CNI nephrotoxicity is considered to be irreversible. The proposed pathways of CNI nephrotoxicity are summarized in Figure 1. For an extensive review of CNI induced nephrotoxicity, see Ref. (3).

Attempts to prevent or reduce CNI nephrotoxicity in humans have focused on angiotensin antagonism or vasodilating agents. Although central in the hypothesized mechanism of CNI nephrotoxicity, studies targeting the effects of angiotensin II have not yielded the expected results on long-term allograft survival. One randomized clinical trial (RCT), although designed to evaluate the effect of angiotensin converting enzyme inhibitor (ACEI) ramipril on cardiovascular outcomes in renal transplant patients, did not show any difference in long-term renal function when compared to placebo (6). Similarly, the angiotensin receptor blocker (ARB) losartan did not have an effect on the composite endpoint of interstitial volume expansion and end-stage renal disease in 153 renal transplant patients after 5 years (7). Early studies indicated a beneficial effect of calcium channel antagonists in both short- (8) and long-term renal allograft function (9, 10); however, results have been somewhat conflicting [summarized in Ref. (3)] and have not translated into clinical practice. Whether the beneficial effect of calcium antagonists on renal function is mainly due to pre-renal factors or due to reduced renal fibrosis remains to be investigated. Studies of nitric oxide (NO) donors or vasodilatory prostanoids in humans and animal studies of anti-transforming growth factor β (TGF-β), antioxidants, statins, and magnesium have not shown a beneficial effect on kidney function (3). An alternative way to reduce CNI nephrotoxicity is *via* CNI minimization or complete CNI withdrawal; however, the majority of attempts have resulted in higher acute rejection rates (11). Of interest are the belatacept-protocols, showing superior graft function with belatacept for 7–10 years when compared with CsA despite higher rates of early acute rejection in the belatacept groups (12, 13). Adverse event rates were similar (12). The use of belatacept as an alternative to CNI in solid organ transplantation has been summarized in a recent review (14).

The relative contribution of CNI nephrotoxicity to late allograft failure has been the object of debate in recent years (15). Early reports indicated a prevalence of chronic CNI nephrotoxicity of almost 100% in renal allograft biopsies after 10 years (16), which was supported by the finding of IF/TA in a large proportion of kidney biopsies from non-renal transplant patients (17). Since then, standard therapy has changed from high dose CsA toward lower-dose tacrolimus (18). Induction therapy in combination with mycophenolate has made CNI minimization possible. A recent study by Nankivell et al. compared sequential kidney graft biopsies from the CsA era (1988–1998) with the tacrolimus era (1999–2012). These showed a lower prevalence of chronic histological lesions in the tacrolimus group, indicating lower nephrotoxicity of current protocols. However, both cellular and humoral acute rejection rates were significantly lower in the tacrolimus era (19) where mycophenolate had also replaced azathioprine. Hence, the superior results of the tacrolimus era might be more complex than merely CNI minimization and reflect both immunological and non-immunological advances.

## Aldosterone and Kidney Fibrosis

**Figure 2:**
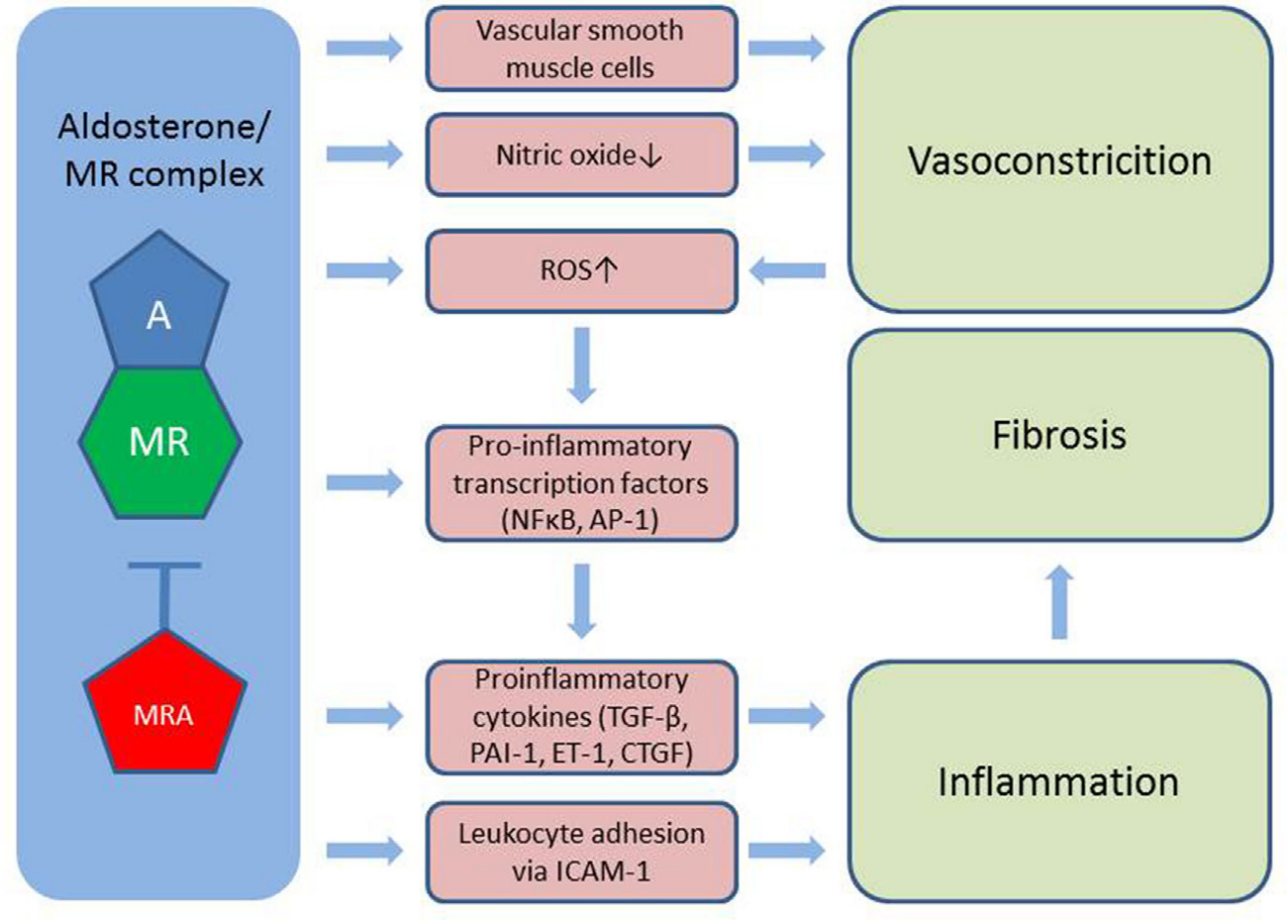
Aldosterone induces vasoconstriction *via* MR in vascular smooth muscle cells and through reduced bioavailability of nitric oxide. Also, aldosterone stimulates the formation of ROS further worsened by vasoconstriction. Activation of pro-inflammatory transcription factors as well as the direct stimulation of cytokines and leukocyte adhesion to the vessel wall leads to inflammation, which contributes to tissue fibrosis. A, aldosterone; MR, mineralocorticoid receptor; MRA, MR antagonist; ROS, reactive oxygen species; TGF-β, transforming growth factor β; PAI-1, plasminogen activator inhibitor 1; ET-1, endothelin 1; CTFG, colony transforming growth factor; ICAM-1, intercellular adhesion molecule 1; NFκβ, nuclear factor κβ; AP-1, activator protein 1.

Aldosterone regulates sodium and water balance *via* the MR in the kidney but is also involved in deleterious processes leading to fibrosis including vasoconstriction, inflammation, and oxidative stress (Figure 2). Upon aldosterone binding to the MR, the receptor translocates to the nucleus where it regulates gene transcription (20). To date, two steroidal MR antagonists have been approved for clinical use, namely spironolactone and eplerenone. Recommended dosages for both drugs are 25–50 mg daily in heart failure and up to 400 mg daily for spironolactone in hyperaldosteronism and liver cirrhosis with ascites. Spironolactone competitively binds to the MR with a high affinity, but also exerts antiandrogenic side effects through binding to the progesterone and androgen receptors (21). Eplerenone is more selective to the MR with fewer antiandrogenic side effects, but simultaneously has a lower affinity for the MR, hence is less potent (22). Much effort has gone into developing newer compounds with high affinity and high selectivity. The non-steroidal MR antagonist finerenone is currently undergoing phase III clinical trials in heart failure and diabetic kidney disease. Further, several other MR antagonists are in the pipeline (23).

Mineralocorticoid receptors are expressed in endothelial- and vascular smooth muscle cells, cardiomyocytes, mesangial cells, and podocytes in the kidney, adipocytes and a number of circulating cells, e.g., monocytes, macrophages, and dendritic cells (24). Aldosterone induces vasoconstriction, particularly under conditions of endothelial damage, likely through a direct action on vascular smooth muscle cells (25), and further reduces bioavailability of the potent vasodilator NO through MR-dependent mechanisms (26). This imbalance between vasoconstriction and vasodilation is commonly referred to as endothelial dysfunction and is associated with increased cardiovascular morbidity and mortality (25). MR antagonism has shown promising results in large scale RCTs in congestive heart failure (27, 28) reducing both morbidity and all-cause mortality. In addition to endothelial dysfunction, aldosterone stimulates the generation of reactive oxygen species by increasing the activity of nicotinamide adenine dinucleotide phosphate oxidase and by reducing the expression of glucose-6-phosphate dehydrogenase. This leads to the formation of superoxide and hydrogen peroxide, which in turn activate pro-inflammatory transcription factors nuclear factor kappa B (Nfκβ) and activator protein 1. Also, the aldosterone/MR complex directly stimulates pro-inflammatory growth factors and cytokines [e.g., TGF-β, plasminogen activator inhibitor 1 (PAI-1), endothelin 1, and colony transforming growth factor (CTGF)]. MR located in endothelial cells stimulates expression of the intercellular adhesion molecule 1, which facilitates the adhesion of leukocytes to the endothelium (24). The inflammatory environment hereby created plays a major part in initiating fibrogenesis (29).

Several clinical studies have evaluated the use of MR antagonists in chronic kidney disease. Results have been summarized in two systematic reviews including 27 and 19 studies, respectively, comprising 33 different trials. Most trials investigated the addition of MR antagonists to ACEI or ARB. Currie et al. reported a reduction of protein excretion of 38.7 ± 21.5% in any measure of protein/albumin excretion including all 19 trials associated with MR antagonism, which could be explained by a reduction of blood pressure (BP) (30). Bolignano et al. reported significantly reduced protein excretion rates for both spironolactone and eplerenone groups in addition to ACEI/ARB when compared to placebo (31). None of the included studies were powered to evaluate the effect on long term kidney function.

## CNIs and the Renin-Angiotensin-Aldosterone System (RAAS)

Cyclosporine A directly stimulates renin synthesis in the juxtaglomerular apparatus in rats and humans and further increases plasma renin activity (PRA) in rats, but this finding has not been reproduced in humans (32). On the contrary, early reports indicated that CsA reduced PRA and did not alter aldosterone levels in both healthy controls (33) and renal transplant patients (34). This difference between rats and humans could reflect physiological differences between species, but could also be related to differences in dosage and duration of treatment (32). A recent RCT measured PRA and plasma aldosterone at baseline and annually for 5 years in kidney transplant patients randomized to losartan or placebo. The majority of PRA and plasma aldosterone measurements in both groups were within normal range. Interestingly, higher aldosterone levels were associated with inferior graft function (35). It has been hypothesized that intrarenal RAAS contributes to CNI nephrotoxicity (32). Eplerenone reduces fibrosis and renal mRNA expression of collagen, TGF-β, and PAI-1 in adrenalectomized rats, indicating an influence of locally produced aldosterone (36). The importance of intrarenal activation of RAAS needs further investigation. As outlined in Figures 1 and 2, many deleterious processes related to aldosterone are represented in the chain of events leading to acute and chronic CNI nephrotoxicity explaining the rationale to investigate the effect of MR antagonism in CNI nephrotoxicity.

## Effects of MR Antagonism in Animal Models of CNI Nephrotoxicity

### Acute CNI Nephrotoxicity

Acute CNI nephrotoxicity is characterized by vasoconstriction of the afferent arteriole leading to a decreased renal blood flow (RBF) and subsequently reduced glomerular filtration. Several groups have demonstrated an effect of MR antagonism preventing the reduction of RBF following CsA (Table 1). Perez-Rojas et al. demonstrated a reduction of RBF in CsA-treated rats. This effect was abolished by spironolactone, which also increased glomerular filtration rates (GFR) (37). The beneficial effect of spironolactone on kidney function in acute CNI nephrotoxicity was already highlighted in the 1980s (38–40). Similar results have been achieved using eplerenone (41). Recent evidence points toward MR in vascular smooth muscle cells to play a role in the vasoconstriction associated with acute CNI nephrotoxicity (42). Regarding structural changes related to acute CNI nephrotoxicity, Nielsen et al. demonstrated a reduction of tubular hyaline vacuolization and arteriolar depositions by eplerenone (41). Also, McAuley et al. found that spironolactone significantly decreased urinary tubular damage marker *N*-acetyl-beta-d-glucosaminidase (38).

**Table 1:** Included animal studies.

Reference	Acute/chronic nephrotoxicity (AN/CN)	Number of rats per group (*n*)	Intervention	Duration	Renal blood flow	GFR	Blood pressure	Proteinuria	Histology	Biomarker altered
McAuley et al. (38)	AN	6	Spiro 25 mg/kg/day	14 days	NI	↑	NI	NI	Less tubular damage	NAG↓
Nielsen et al. (41)	AN	7	EPL 100 mg/kg/day	21 days	↑	↑	↓	NI	Less hyaline vacuolization and vascular depositions	NI
Perez-Rojas et al. (37)	AN/CN	10/6	Spiro 20 mg/kg/day	7 days/21 days	↑	↑	NI	NI	NI	Prorenin↓, AT2↓, ETb↓
Feria et al. (43)	CN	12	Spiro 20 mg/kg/day	21 days	NI	↑	→	→	Less fibrosis and arteriolopathy	TGFβ↓, collagen I↓, collagen IV↓, fibronectin↓, EGF↑
Perez-Rojas et al. (48)	CN	7	Spiro 20 mg/kg/day	36 days	NI	↑	→	NI	Less fibrosis, arteriolar thickening, glomerular constriction, and apoptosis	Procaspase-3↓, TGFβ↓, KIM-1↓
Macunluoglu et al. (44)	CN	8	Spiro 20 mg/kg/day	28 days	NI	↑	NI	→	Less fibrosis	TGFβ↓, PDGF↓
Silva et al. (46)	CN	6	Spiro 20 mg/kg/day	35 days	NI	→	NI	→	No effect	NI
Waanders et al. (47)	CN	8	Spiro 20 mg/kg/day	12 weeks	NI	→	→	→	No effect	NI
Nielsen et al. (45)	CN	11	EPL 100 mg/kg/day	12 weeks	NI	↑	↓	NI	Less fibrosis, glomerulo-sclerosis, hyaline tubular casts, tubular dilatation, and hyaline arteriolopathy	E-cadherin↑
Sun et al. (36)	CN	8	EPL 100 mg/kg/day	4 weeks	NI	→	NI	↓	Less fibrosis	Collagen I↓, TGFβ↓, PAI-1↓

*The effect of mineralocorticoid receptor antagonism is indicated by (↑): increase (→): no change or (↓): decrease*.

*AN, acute calcineurin inhibitor (CNI) nephrotoxicity, CN, chronic CNI nephrotoxicity, Spiro, spironolactone; EPL, eplerenone; NI, not investigated; NAG, *N*-acetyl-beta-d-glucosaminidase; AT2, angiotensin 2; ETb, endothelin b receptor; TGFβ, transforming growth factor β; EGF, epithelial growth factor; KIM-1, kidney injury molecule 1; PDGF, platelet-derived growth factor; PAI-1, plasminogen activator inhibitor 1*.

### Chronic CNI Nephrotoxicity

Several aspects of chronic CNI nephrotoxicity in rat models have been investigated. Most studies initiate MR antagonists in parallel with CsA treatment, thus investigating if chronic kidney damage can be prevented (36, 37, 43–47). One study, however, investigated whether MR antagonism was beneficial after development of IF/TA. This showed improvement of GFR, fibrosis, and apoptosis, while all parameters further deteriorated in the non-MR antagonist group (=CsA-group) (48).

#### Renal Blood Flow

Similar to acute CNI nephrotoxicity, CsA has been shown to reduce RBF in rat models of chronic CNI nephrotoxicity, as indirectly indicated by constriction of glomeruli. This constriction was partly reverted by spironolactone (48). The molecular background for the vasoconstriction was investigated in sodium-depleted rats (37). They found an upregulation of prorenin by CsA, reverted by spironolactone. This study further demonstrated that the addition of spironolactone resulted in downregulation of the renal expression of endothelin A receptors and upregulation of endothelin B receptors, favoring vasodilation. Spironolactone had no influence on endothelin expression, adenosine pathways, and cyclooxygenase 2 mRNA levels (37). Taken together, this indicates that the beneficial effect of MR antagonism on renal vasoconstriction in chronic CNI nephrotoxicity involves both RAAS and endothelin pathways.

#### Glomerular Filtration Rate

The majority of studies on chronic CNI nephrotoxicity in rats demonstrate a beneficial effect of MR antagonism on GFR (37, 43–45, 48). This relates to reduced renal vasoconstriction, but a likely contribution is the reduction of structural changes. One study failed to reach significance evaluating GFR based on s-creatinine levels after 5 weeks of spironolactone (46). In this study, blood concentrations of CsA were substantially lower than in similar studies and all kidneys were morphologically normal.

To our knowledge, only one study has investigated the effect of MR antagonsim in a model of renal transplantation in rats (47). In this study, 16 rats had kidney grafts from the same strain (Dark agouti—isografted) and 14 rats had kidney grafts from a different strain (Dark Agouti to Wistar Furth—allografted). All animals received CsA and were randomized to spironolactone 20 mg/kg/day or vehicle. After 12 weeks, there was no difference in GFR between vehicle and spironolactone groups in both iso- and allografted animals as evaluated by creatinine clearance. The allograft model was developed to mimic the histologic changes seen in chronic rejection in humans (49), thereby reflecting both immunologic and non-immunologic allograft damage. The observation that there was no fibrosis in kidneys from isografted animals despite CsA-treatment and extensive fibrosis in allografted animals highlights an immunological fibrogenesis rather than induced by CsA.

#### Blood Pressure

In contrast to CNI treatment in humans, rat models of CNI nephrotoxicity are not generally associated with hypertension. Accordingly, the majority of studies in chronic CNI nephrotoxicity did not find any significant effect of neither CNI treatment nor MR antagonist on BP by invasive (48) or non-invasive measurements (43, 47). One group, however, did find a significant increase in both daytime and nighttime BP in the CsA group compared to controls evaluated by intraarterial measurements in conscious animals. Eplerenone reduced BP during the resting period (45).

#### Proteinuria

Only one of the reported studies of chronic CNI nephrotoxicity showed reduced proteinuria during MR antagonism (36). One study even reported a tendency for a lower grade of proteinuria in the CsA group compared to vehicle and spironolactone groups (43). This observation could be partly explained by the simultaneous reduction of GFR but could also reflect differences between rats and humans.

#### Fibrosis

Feria et al. found less tubulointerstitial fibrosis in the spironolactone group (43). This was later confirmed by Macunluoglu et al. with all samples in the CsA-group displaying moderate to severe interstitial fibrosis (>25% affected area) compared to only one-fourth of samples in the spironolactone-group (44). Nielsen et al. showed increased volume fraction of renal interstitium in the CsA-group compared to controls, which was reduced in the eplerenone-group (45). Perez-Rojas et al. investigated the effect of MR antagonism initiated after established chronic CNI nephrotoxicity. After 18 days of CsA treatment, kidney sections from six rats displayed 20.2% tubulointerstitial fibrosis. Interestingly, spironolactone, when initiated at day 18, seemed to slow the progression of fibrosis at day 36 compared to CsA alone (48). Feria et al. and Nielsen et al. further demonstrated less arteriolopathy in groups treated with MR antagonists compared with CsA (43, 45).

#### Markers of Kidney Damage

In addition to histological changes in chronic CNI nephrotoxicity, focus has been on identifying markers of fibrosis. The molecular regulation of the fibrotic processes is an intricate cascade of cytokines and growth factors leading to increased transcription of constituents of fibrosis, i.e., collagen I, III, and fibronectin. CsA increases several fibrogenic markers in models of chronic CNI nephrotoxicity. One of the major pro-fibrotic cytokines is TGF-β acting *via* (1) the canonical pathway to activate transcription factors Smad2 and Smad3, which, in complex with Smad4, act to induce the transcription of pro-fibrotic molecules or (2) the non-canonical, Smad-independent pathways activating mitogen-activated protein kinases, c-Jun terminal kinase, p38, and extracellular-signal regulated kinase to increase the transcription of other pro-fibrotic cytokines as well as collagens and fibronectin (50, 51). Several studies have demonstrated increased renal mRNA levels of TGF-β in response to CsA, an increase that was attenuated by MR antagonsim (36, 43, 48). Macunluoglu et al. demonstrated a similar effect of MR antagonists by immunostaining of renal tissue (44).

In accordance with findings regarding TGF-β, CsA increased renal mRNA levels of both collagen I (36, 43) and fibronectin (43). This increase was mitigated by MR antagonism. CsA also increased α-smooth muscle actin—a marker of activated fibroblasts, although these levels were independent of MR antagonism (45), and the renal mRNA levels of several other profibrotic cytokines, i.e., platelet-derived growth factor (PDGF) (44), CTGF, and PAI-1 (36) all of which participate in the profibrotic cascade involving TGF-β. Concomitant MR antagonism significantly reduced levels of PDGF (44), and PAI-1, but not CTGF (36).

Tubular atrophy associated with renal fibrosis is partly due to apoptosis of tubular epithelial cells. Perez-Rojas et al. found a significant reduction of apoptotic nuclei in renal cortex by addition of spironolactone to CsA. This was paralleled by a reduction of apoptosis-marker procaspase 3 in the spironolactone group. Also, they demonstrated an increase in both cortical and urinary mRNA levels of kidney injury molecule 1 (KIM-1) in response to CsA and subsequent reduction by spironolactone (48).

Epithelial growth factor (EGF) stimulates regeneration of the tubular epithelium after ischemic damage and Feria et al. found a significant reduction of cortical EGF caused by CsA. Spironolactone diminished this reduction when added to CsA, but interestingly when administered alone also significantly reduced cortical EGF levels compared with vehicle (43). Nielsen et al. demonstrated a reduction of the epithelial marker E-Cadherin in response to CsA, which was attenuated by eplerenone (45). Taken together, this indicates a protective effect of MR antagonists on tubular epithelial cells. Whether this is mainly due to prevention of TGF-β-induced apoptosis and the reduction of ischemic damage to the kidney as a result of higher RBF remains to be established. None of the identified animal studies have addressed the potential effect of MR antagonism in reducing inflammation and endothelial dysfunction. These pathways may contribute to the renoprotection conferred by MR antagonists. For an overview of the included studies, see Table 1.

## Effects of MR Antagonists in Renal Transplant Patients

Despite evidence from animal studies, very few studies have investigated the effect of MR antagonists preventing CNI nephrotoxicity in humans. This is partly due to concerns regarding hyperkalemia related to MR antagonism in patients with impaired renal function and concomitant CNI treatment. This issue was addressed in a recent publication testing the safety of eplerenone 25 mg/day for 8 weeks in a group of 31 kidney transplant patients treated with CsA. Median eGFR was 41 mL/min. A slight increase in serum-potassium was detected already after 2 days of treatment. Nine patients had at least one episode of mild hyperkalemia (>5.0 mmol/L) but only one patient experienced moderate hyperkalemia (>5.5 mmol/L). No patients were withdrawn from the study due to hyperkalemia. There was no change in systolic BP, body weight, or s-bicarbonate after 8 weeks of treatment (52).

One single-blind RCT has investigated the effect of MR antagonism in a pediatric kidney transplant population. Twenty-four children with chronic allograft nephropathy were randomized to eplerenone or placebo for 24 months. Although underpowered to provide definite evidence, there was a tendency toward lower levels of tubulointerstitial fibrosis in the eplerenone group after 24 months (*p* = 0.06), but no significant changes in albuminuria, BP and eGFR. No difference was detected in urinary KIM-1, heat-shock protein 72 (HSP-72), 8-hydroxy-2-deoxyguanosine (marker of oxidative stress), or serum TGF-β (53).

The effect of MR antagonism on proteinuria has been addressed in an open label study of 11 proteinuric kidney transplant patients, who already received both ACEI and ARB. This study found a significant reduction in protein excretion from 4.4 to 2.7 g/day after the first month of treatment with spironolactone 25 mg/day, an effect that was sustained for the full 6 months of intervention. There was no difference in BP or GFR during this period (54).

The proposed effect of MR antagonists to reduce renal ischemia and thereby oxidative stress was investigated in a double-blind RCT including 20 kidney transplant recipients randomized to spironolactone 25 mg or placebo on the day before transplantation and 3 days after. The aim was to investigate the effect of MR antagonism on oxidative stress relating to ischemia/reperfusion injury. Urinary hydrogen peroxide (H_2_O_2_) levels were significantly lower in the spironolactone group on the fifth postoperative day. There was no difference in tubular damage markers KIM-1, interleukin 18, or HSP-72. However, three patients in the spironolactone group displayed delayed graft function and subsequent biopsies revealed acute rejection in two of these. In the placebo group, one patient had rejection. It is worth noticing that B-Tac trough levels were possibly lower in the spironolactone group (3.65 vs. 6.05 ng/mL), although this was not significant. A difference in B-tacrolimus concentration could account for the greater rejection rate in the spironolactone group, but could also contribute to lower H_2_O_2_ levels, since tacrolimus reduces RBF, thereby inducing oxidative stress (55). The included human studies are summarized in Table 2.

**Table 2:** Included studies in renal transplant patients.

Reference	Number of subjects	Intervention	Design	Result
Bertocchio et al. (52)	31	EPL 25 mg/day for 8 weeks	Prospective, open-label	No significant hyperkalemia

Medeiros et al. (53)	24	EPL 25 mg/day for 24 months	Prospective, randomized, single-blind, placebo controlled	Stable kidney function
				Tendency toward lower proteinuria and less fibrosis (NS)

Gonzalez Monte et al. (54)	11	Spiro 25 mg/day for 6 months	Prospective, open-label	Significant reduction of proteinuria

Ojeda-Cervantes et al. (55)	20	Spiro 25 mg/day 24 h prior to kidney transplantation and 3 days after	Randomized, double blind, placebo controlled	Reduced urinary H_2_O_2_
				No difference in tubular damage markers

*EPL, eplerenone; Spiro, spironolactone; NS, not significant; H_2_O_2_, hydrogen peroxide*.

## Current and Future Trials

Evidence underlines the need for sufficiently powered RCTs to evaluate the effect of MR antagonism in the kidney transplant population. Three ongoing trials were identified (https://ClinicalTrials.gov): (I) RCT (NCT02490904) investigating the effect of eplerenone for four days in relation to kidney transplantation in 132 patients on short- and long-term kidney function; (II) open label study (NCT01510795) evaluating the chronic BANFF-scores in 40 patients after 6 months of spironolactone as compared with a retrospective control group, and (III) RCT investigating the effect of spironolactone for 3 years in 170 patients with regards to preserving kidney function and reducing renal fibrosis (NCT01602861).

## Conclusion and Perspectives

Calcineurin inhibitor causes renal fibrosis likely due to altered hemodynamics and a direct stimulatory effect on fibrogenic factors. Rat models of acute and chronic CNI nephrotoxicity have shown beneficial effects of MR antagonism in preserving GFR and reducing fibrosis. This is likely due to an increased RBF attenuating ischemic damage to the kidneys, in combination with a direct inhibitory action on pro-fibrotic cytokines. Few human studies have addressed this hypothesis. Improving long-term allograft survival remains a key challenge in renal transplantation with the potential to improve quality of life, morbidity, and mortality of the affected individuals. Results of future and ongoing clinical trials may clarify if aldosterone antagonism can prevent or improve CNI nephrotoxicity in humans.

## Author Contributions

LM wrote the article, CB and HT reviewed and revised the article.

## Conflict of Interest Statement

The authors declare that the research was conducted in the absence of any commercial or financial relationships that could be construed as a potential conflict of interest.

## Abbreviations

ACEI, angiotensin converting enzyme inhibitors; ARB, angiotensin receptor blockers; BP, blood pressure; CNI, calcineurin inhibitor; CsA, cyclosporine A; CTGF, colony transforming growth factor; EGF, epithelial growth factor; GFR, glomerular filtration rate; H_2_O_2_, hydrogen peroxide; HSP-72, heat-shock protein 72; ICAM-1, intercellular adhesion molecule 1; IF/TA, interstitial fibrosis and tubular atrophy; KIM-1, kidney injury molecule 1; MR, mineralocorticoid receptor; NADP, nicotinamide adenine dinucleotide phosphate; NFAT, nuclear factor of activated T-cells; Nfκβ, nuclear factor kappa B; PAI-1, plasminogen activator inhibitor 1; PDGF, platelet-derived growth factor; PRA, plasma renin activity; RAAS, renin-angiotensin-aldosterone system; RBF, renal blood flow; RCT, randomized clinical trial; TGF-β, transforming growth factor β.
